# The effect of extreme spring weather on body condition and stress physiology in Lapland longspurs and white-crowned sparrows breeding in the Arctic

**DOI:** 10.1016/j.ygcen.2016.07.015

**Published:** 2016-10-01

**Authors:** Jesse S. Krause, Jonathan H. Pérez, Helen E. Chmura, Shannan K. Sweet, Simone L. Meddle, Kathleen E. Hunt, Laura Gough, Natalie Boelman, John C. Wingfield

**Affiliations:** a Department of Neurobiology, Physiology and Behavior, University of California Davis, One Shields Avenue, Davis, CA 95616, USA; b Department of Earth and Environmental Sciences, and Lamont-Doherty Earth Observatory of Columbia University, Palisades, NY 10964, USA; c The Roslin Institute, The Royal (Dick) School of Veterinary Studies, The University of Edinburgh, Easter Bush, Midlothian EH25 9RG, Scotland, UK; d John H. Prescott Marine Laboratory, Research Department, New England Aquarium, Boston, MA 02110, USA; e Department of Biological Sciences, Towson University, Towson, MD 21252, USA

**Keywords:** Corticosterone, Hypothalamic-pituitary-adrenal (HPA) axis, Climate change, Life history trade-offs

## Abstract

Climate change is causing rapid shifts in temperature while also increasing the frequency, duration, and intensity of extreme weather. In the northern hemisphere, the spring of 2013 was characterized as extreme due to record high snow cover and low temperatures. Studies that describe the effects of extreme weather on phenology across taxa are limited while morphological and physiological responses remain poorly understood. Stress physiology, as measured through baseline and stress-induced concentrations of cortisol or corticosterone, has often been studied to understand how organisms respond to environmental stressors. We compared body condition and stress physiology of two long-distance migrants breeding in low arctic Alaska – the white-crowned sparrow (*Zonotrichia leucophrys*) and Lapland longspur (*Calcarius lapponicus*) – in 2013, an extreme weather year, with three more typical years (2011, 2012, and 2014). The extended snow cover in spring 2013 caused measureable changes in phenology, body condition and physiology. Arrival timing for both species was delayed 4–5 days compared to the other three years. Lapland longspurs had reduced fat stores, pectoralis muscle profiles, body mass, and hematocrit levels, while stress-induced concentrations of corticosterone were increased. Similarly, white-crowned sparrows had reduced pectoralis muscle profiles and hematocrit levels, but in contrast to Lapland longspurs, had elevated fat stores and no difference in mass or stress physiology relative to other study years. An understanding of physiological mechanisms that regulate coping strategies is of critical importance for predicting how species will respond to the occurrence of extreme events in the future due to global climate change.

## Highlights

• The spring of 2013 was extreme with record low temperatures and snow cover.
• Arrival of migrant birds in Arctic was significantly delayed in 2013 compared to 3 other years.
• Body condition was negatively affected in white-crowned sparrows and Lapland longspurs.
• Stress physiology was increased in Lapland longspurs but not white-crowned sparrows.
• Extreme events have the capacity to affect phenology, body condition and stress physiology.

## 1 Introduction

Flora and fauna have evolved physiological mechanisms to cope with environmental variation within a predictable range (Post et al., 2009, Helm et al., 2013). As climate change is causing rapid changes in temperature regimes around the globe (Meehl et al., 2007), warming temperatures have advanced spring events in recent years (Parmesan, 2007). Existing evidence indicates spring temperatures and snow cover are important predictors of phenology for plants (Sweet et al., 2014, Bjorkman et al., 2015), arthropods (Høye et al., 2007, Tulp and Schekkerman, 2008), birds (Glądalski et al., 2014, Visser et al., 2015), and mammals (Post et al., 2009, Helm et al., 2013, Sheriff et al., 2013). Therefore, it is of critical importance to understand how flora and fauna will cope with rapid climate change both through changes in phenology and physiology (Parmesan, 2007, Wingfield et al., 2015).

Rapid changes in temperature have been accompanied by an increase in the frequency, intensity and duration of unpredictable weather events (Easterling et al., 2000, Smith, 2011). Extreme events, by definition, represent just 5% of recorded weather patterns and can vary in duration from single day to several months (Smith, 2011). The year 2013 was characterized by extreme spring weather conditions that lasted several months with record low temperatures and persistent snow cover affecting much of Europe, North America, and Asia (Screen and Simmonds, 2010, Glądalski et al., 2014). Snow cover in Europe in March of 2013 was the highest it has been in the last 400 years (Glądalski et al., 2014). Similarly, in northern North America, snow cover persisted unusually late into the season, breaking decade long records, while temperatures were some of the coldest recorded in the last century (Stuefer et al., 2013, Lee et al., 2015). Profound phenological consequences of this extremely cold spring have already been reported across taxonomic groups (Glądalski et al., 2014, Sweet et al., 2014, Lee et al., 2015, Senner et al., 2015, Boelman et al., in review). Such harsh conditions can be energetically challenging for birds and cause declines in body condition (Sandberg and Moore, 1996, Newton, 2006), but little is known about the changes in physiology and body condition in response to extreme events.

Physiological changes must occur for an individual to survive in conditions that are beyond those normally experienced (Boonstra, 2004, Wingfield et al., 2015). The role of the hypothalamic-pituitary-adrenal (HPA) axis in modifying vertebrate behavior and physiology in response to unpredictable events has been studied extensively (Sapolsky et al., 2000). In response to stressful events such as storms, predation, food shortages, or cold temperatures, the HPA axis in birds is activated through a series of neuroendocrine and endocrine signaling mechanisms that result in the production of the stress hormone corticosterone (Sapolsky et al., 2000, Boonstra, 2004). The activity of the HPA axis, which can be assessed by measuring baseline or stress-induced corticosterone concentrations, increases as conditions become harsher (Wingfield et al., 1983, Rogers et al., 1993, Smith et al., 1994, Astheimer et al., 1995, Raouf et al., 2006, Jenni-Eiermann et al., 2008, Krause et al., 2015a, Krause et al., 2016a, Walker et al., 2015). In the short-term, baseline concentrations of corticosterone are important for regulating behavior and physiology such as the catabolism of fat and protein, which can ultimately lead to changes in body condition (Breuner and Hahn, 2003, Landys et al., 2006). Over the longer term, stress-induced increases in circulating corticosterone help mediate life-history trade-offs because they direct resources away from the current life history stage (e.g., breeding, migration, molt) towards self-preservation (Wingfield et al., 1998, Krause et al., 2015c). Given these trade-offs between current and future fitness, the degree to which the HPA axis is activated during an extreme event should, in theory, match the level of the disturbance (Angelier and Wingfield, 2013, Wingfield et al., 2015).

The goal of this study was to understand how the physiology and body condition of migrant songbirds respond to changes in environmental conditions across breeding seasons. Over four consecutive years, we monitored two species of long-distance migrant passerines: the shrub-nesting white-crowned sparrow (*Zonotrichia leucophrys gambelii*) and the tussock-tundra-nesting Lapland longspur (*Calcarius lapponicus*) breeding in low arctic Alaska, USA. In 2013, extreme conditions delayed both arrival and clutch initiation of both species relative to the other three study years (Boelman et al., in review). To our knowledge, there is little published evidence on the effects of prolonged extreme conditions, like those experienced in 2013, on physiology and body condition indices (Martin and Wiebe, 2004). In birds, body condition is often assessed using measures of subcutaneous fat stores, pectoralis muscle profile, body mass and hematocrit level (Kaiser, 1993, Fair et al., 2007, Krause et al., 2015a). We hypothesized that prolonged extreme environmental conditions experienced in 2013 would reduce overall body condition (fat stores, body mass, pectoralis muscle profile, and hematocrit) and increase the activity of the HPA axis (assessed by both at baseline and stress-induced corticosterone concentrations).

## 2 Study site and species

**Fig. 1:**
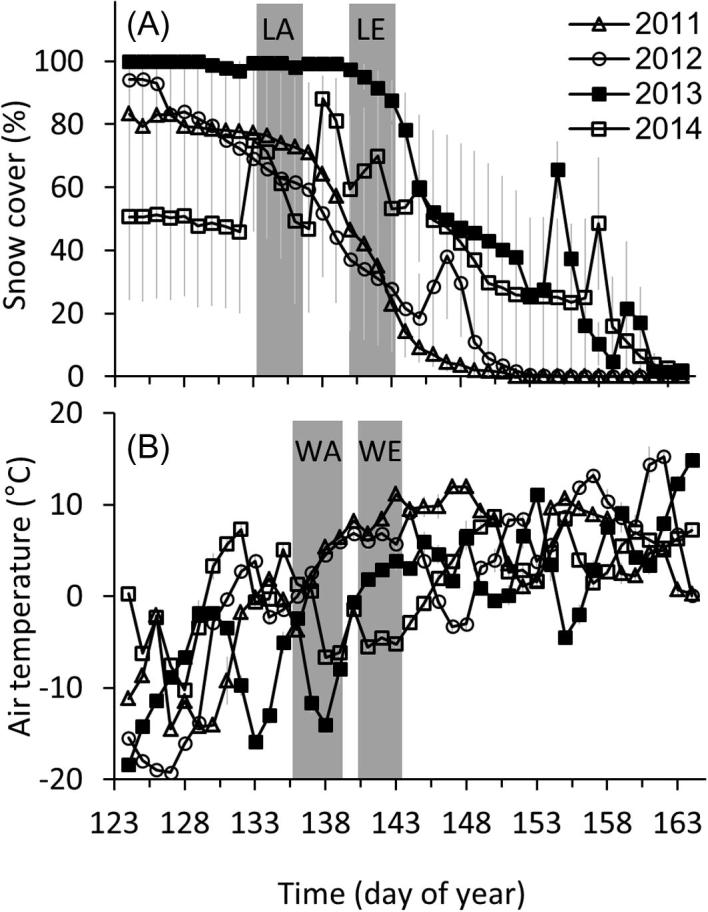
Daily (A) snow cover and (B) average daily temperatures from early-May to mid-June. Mean arrival dates (shaded in gray) in an average year were Julian day 136 for Lapland longspurs (LA) and 138 for white-crowned sparrows (WA) caught in 2011, 2012, and 2014. Arrival dates were delayed in the extreme year of 2013 with the mean arrival date of Julian day 141 for Lapland longspurs (LE) and 142 for white-crowned sparrows (WE), respectively. Snow cover was highest during the arrival period in 2013 compared to the other years. See Boelman et al. (in review) for more details.

This study was conducted from 2011 to 2014 at Toolik Field Station, located in the foothills of the Brooks Range on the North Slope of Alaska, USA (N 68° 38′, W 149° 36′). Lapland longspurs and Gambel’s white-crowned sparrows are long-distance migrant songbirds that winter in the contiguous United States and migrate annually to their breeding grounds at higher latitudes (Custer and Pitelka, 1977, Boelman et al., 2015). Lapland longspurs are arctic-breeding specialists with a circumpolar breeding distribution from the Low to the High Arctic and prefer to nest on tussock and polygon tundra (Custer and Pitelka, 1977). Gambel’s white-crowned sparrows breeding range does not extend above the Low Arctic as this species prefers to nest on tundra dominated by deciduous woody shrubs and evergreens; they are thought to be more recent colonizers of the Arctic (Boelman et al., 2015, Krause et al., 2015a). Arrival dates were determined by daily surveys, initiated prior to the arrival of the birds in early May, conducted both along the Dalton Highway and at each of our four field sites: Roche Mountonee Creek (N 68° 22′, W 149° 18′), Imnavait Creek (68° 37′, W 149° 17′), Sagavanirktok Department of Transportation (68° 45′, W 148° 53′), and Toolik Field Station (N 68° 38′, W 149° 36′). Lapland longspurs typically arrive on the breeding grounds in early May, while Gambel’s white-crowned sparrows arrive in mid-May (see Fig. 1; Boelman et al., in review).

## 3 Weather and snow cover

Weather data including temperature (°C), precipitation (mm), and wind speed (m/s) were collected by meteorological stations at each site (Environmental Data Centre, 2014). Snow cover was characterized by time-lapse images and analyzed for percent cover using ImageJ as previously described by Krause et al. (2016b).

## 4 Capture and blood sampling

**Fig. 2:**
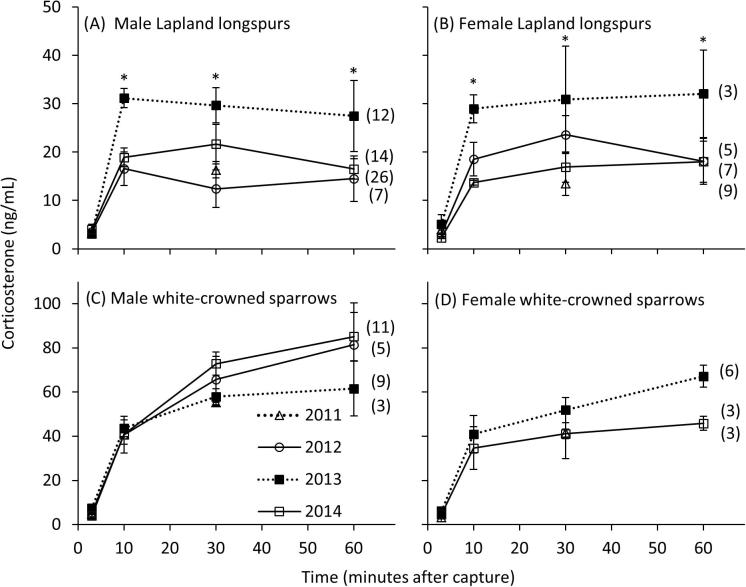
The effect of year on corticosterone concentrations in response to acute restraint stress in (A) male and (B) female Lapland longspurs and (C) male and (D) female white-crowned sparrows. Four point sampling was used from 2012 to 2014 (0, 10, 30, and 60 min) while 2011 utilized two point sampling (0 and 30 min). There was a significant effect of year on corticosterone concentrations in Lapland longspurs but not white-crowned sparrows. There was a significant effect of sex on corticosterone levels in white-crowned sparrows, only. The numbers in parentheses indicate sample size for each group. Values presented as mean ± SEM. ^*^P < 0.05.

**Fig. 3:**
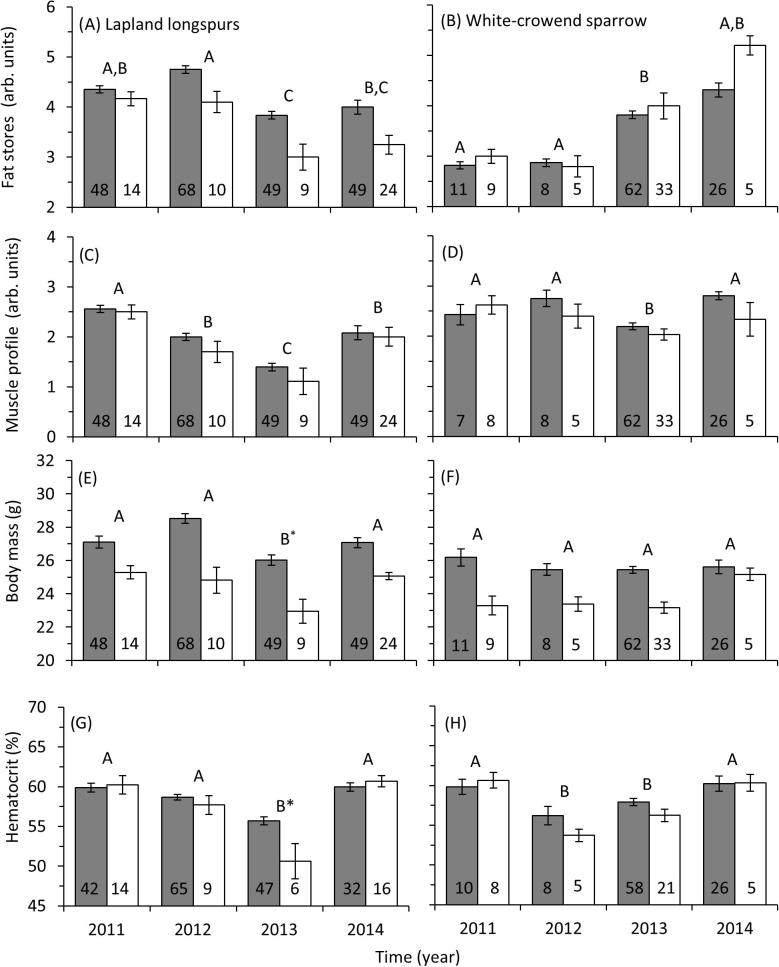
The effect of year on total fat stores, pectoralis muscle profile, body mass and hematocrit levels in male (gray) and female (white) Lapland longspurs (left panels: A, C, E, G) and white-crowned sparrows (right panels: B, D, F, H). Lapland longspurs had reduced values for all comparisons in 2013. White-crowned sparrows had lower hematocrit levels and pectoralis muscle profiles in 2013 while fat stores were higher than 2011 and 2012. Samples sizes for each group are indicated in the bars for each graph. Letters that are different from one another indicate significant differences between years while an asterisk indicates a difference between males and females (Tukey’s HSD *P* < 0.05). The numbers within each bar indicate sample size for each group. Values presented as mean ± SEM.

The total number of Lapland longspurs included in the analyses were 73 (for hormones) and 183 (for body condition) and for white-crowned sparrows there were 55 (for hormones) and 152 (for body condition). Specifics for male and female numbers for each species can be found in Fig. 2 for hormonal data and Fig. 3 for body condition data. All birds were caught during the arrival period, lasting approximately 7 days, which included the date on which they first arrived on the breeding grounds until territory establishment (see Boelman et al., in review). Both species were caught with seed-baited potter traps and mist nets, although a greater proportion of Lapland longspurs were caught with traps compared to white-crowned sparrows. HPA axis activity in response to acute restraint stress was measured as previously described (Wingfield et al., 1992). Briefly, the alar vein was punctured with a 26 gauge needle and a baseline blood sample was collected into a heparinized microcapillary tube within 3 min of capture (Romero and Reed, 2005). The mean ± standard deviation for the time to sample from initial capture for Lapland longspurs and white-crowned sparrows were 123 ± 33 and 115 ± 35 s, respectively. Birds were sampled serially at 10, 30 and 60 min post-capture (2012–2014) or only at 30 min post-capture (2011). Birds were held in an opaque cloth bag in between bleeds, and were given a unique set of color bands and an aluminum band from US Geological Survey for later identification in the field. Morphometrics of wing chord, tarsus, beak, body mass, pectoralis muscle profile [0 (emaciated) to 3 (bulging muscle) (Bairlein and Totzke, 1992)], and fat stores for both furcular and abdominal regions [on a scale from 0 (lean) to 8 (fat)] (Kaiser, 1993) were recorded. Blood samples were stored on ice until later processing in the laboratory. Samples underwent centrifugation at 10,000 RPM for 5 min to separate erythrocytes from plasma. Hematocrit was measured for each baseline blood sample, only (Krause et al., 2016a). Plasma was aspirated with a Hamilton syringe and placed into a microcentrifuge tube. Plasma was stored at −80 °C both at the field station, and at the University of California Davis until corticosterone quantification.

## 5 Corticosterone assay

Corticosterone concentrations were quantified using a radioimmunoassay as previously described in detail by Wingfield et al. (1992). In brief, 10 μL of plasma was measured and then 2000 CPM of tritiated corticosterone was added to monitor percent-recoveries (i.e., extraction efficiency) for every sample. Steroids were extracted with 4 mL dichloromethane (redistilled within 24 h of use) for 3 h. Extracts were placed into a water bath at 35 °C, dried under a stream of nitrogen, and then reconstituted using phosphate-buffered saline with gelatine (PBSG). A 100 μL aliquot was added to a scintillation vial and combined with scintillation fluid to determine percent recoveries. Duplicate 200 μL aliquots were assayed by adding 100 μL (∼10^4^ CPM) of tritiated label (Perkin Elmer NET399250UC) and 100 μL of antibody (Esoterix Inc. B3-163). Unbound steroid was stripped from solution by the addition of 500 μL of dextran coated charcoal followed by centrifugation at 3000 RPM. The supernatant was decanted and combined with scintillation fluid (Perkin Elmer Ultima Gold: 6013329) and counted for 6 min or within 2% accuracy on a Beckman 6500 liquid scintillation counter. Final hormone values were corrected using the individual recovery for each sample. Mean recoveries were 84.86% and intra-assay (calculated using C.V. between duplicates) and inter-assay variations were 8.25% and 11.87%, respectively. The mean ± standard error for the detection limits of the assays was 8.87 ± 0.49 pg per tube.

## 6 Statistical analyses

Statistical analyses were performed using JMP 11 Pro (SAS Institute Inc., Cary, NC, 1989–2007). Plasma steroid hormone concentrations were log transformed prior to analysis. All response variables for linear mixed effects models were checked for normality using the Shapiro-Wilks test by plotting the residuals against the predicted value. A linear mixed effects model was tested using a residual covariance structure in which each individual was included as a random variable to test how the dependent variable of hormone concentration was affected by the main effects of acute restraint stress (stress), sex, year, their interactions and the covariates of percent snow cover and mean temperature. All post hoc analyses were performed using Tukey’s Honest Significant Difference (HSD) test. An integrated corticosterone concentration for four point stress series were calculated using the trapezoidal rule, in which baseline samples were subtracted from each time point. A linear mixed effect model was used to investigate the effect of year, sex and their interaction on hematocrit levels, fat stores, pectoralis muscle profile and body mass. Post hoc analyses were performed using Tukey’s HSD test. The relationships between integrated and baseline corticosterone concentrations and hematocrit, fat stores, pectoralis muscle profile, and body mass were investigated using independent linear regression.

## 7 Results

### 7.1 Snow cover

Snow cover during the arrival period differed significantly across years (Fig. 1). Snow cover was higher in 2013 compared to all other study years (Boelman et al., in review). By the time the birds arrived on the breeding grounds, mean daily temperatures were similar to other years of the study (Boelman et al., in review). In 2014, several snowstorms occurred during the latter part of the arrival period.

### 7.2 HPA axis activity

Lapland longspur HPA axis activity increased in response to acute restraint stress and was significantly affected by year and the interaction between year and stress (Table 1, Fig. 2). HPA axis activity was not affected by minimum temperature, percent snow cover, or sex (Table 1). Since the main effect of sex was not statistically significant, post hoc tests were performed on both sexes combined to compare across years. Baseline corticosterone did not differ across years (F_3,80_ = 0.04, *P* = 0.98). In 2013, corticosterone concentrations were higher compared to all other years at the10, 30, and 60 (Tukey’s HSD P < 0.05) minute time points with the exception of 60 min in 2014 (t = 3.18, *P* = 0.07). Integrated corticosterone concentrations were affected by sampling year (F_2,25_ = 7.64, *P* = 0.002) but not by sex (F_1,25_ = 0.45, *P* = 0.50) or the interaction of sex and year (F_2,25_ = 0.85, *P* = 0.43). Integrated corticosterone concentrations were higher in 2013 compared to 2012 (t = 3.63, *P* = 0.003) and 2014 (t = 3.28, *P* = 0.008).

White-crowned sparrow HPA axis activity increased in response to acute restraint stress and was significantly higher in males compared to females (Table 1, Fig. 1). HPA axis activity was not affected by year, percent snow cover, mean air temperature, or the interaction of year and stress (Table 1). Integrated corticosterone concentrations were not different across years in males (F_2,21_ = 1.29, *P* = 0.17) or in females (F_1,5_ = 2.94, *P* = 0.14).

### 7.3 Relationship between HPA axis activity and body condition

For male Lapland longspurs, integrated corticosterone was negatively related to fat stores, pectoralis muscle profile, and mass (Table 2). In female white-crowned sparrows, body mass was also negatively related to integrated corticosterone concentrations (Table 2). However, no significant relationships were found for female Lapland longspurs or male white-crowned sparrows for integrated or baseline corticosterone*.*

### 7.4 Fat stores

Lapland longspur and white-crowned sparrow fat stores were affected by year and sex (Table 3, Fig. 3). Fat stores in Lapland longspurs were significantly lower in 2013 compared to 2011 (t = 2.70, *P* = 0.03) and 2012 (t = 3.21, *P* = 0.008). In white-crowned sparrows, fat stores were higher in 2013 compared to 2011 (t = 3.12, *P* = 0.001) and 2012 (t = 2.12, *P* = 0.03).

### 7.5 Body mass

Lapland longspur body mass was significantly affected by sampling year and sex (Table 3, Fig. 3). For both sexes, body mass was lower in 2013 compared to 2011 (t = 3.29, *P* = 0.006), 2012 (t = 2.17, *P* = 0.004) and 2014 (t = 1.57, *P* = 0.006). On average males (27.1 ± 0.3 g) were heavier than females (24.5 ± 0.15 g) during the arrival period. White-crowned sparrow body mass was significantly affected by sex only (Table 3, Fig. 3). Males (25.5 ± 0.17 g) on average were heavier than females (23.3 ± 0.27 g).

### 7.6 Pectoralis muscle profile

Lapland longspur and white-crowned sparrow pectoralis muscle profiles were significantly affected by sampling year but not by sex or the interaction of sex and year (Table 3, Fig. 3). Since the sexes were not significantly different, muscle profile data were combined to analyze changes across years. Pectoralis muscle profiles in Lapland longspurs were smaller in 2013 compared to 2011 (t = 7.40, *P* < 0.001), 2012 (t = 5.16, *P* < 0.001), and 2014 (t = 4.23, *P* < 0.001). Muscle profiles in white-crowned sparrows were lower in 2013 compared to 2011 (t = 6.95, *P* = 0.01), 2012 (t = 2.90, *P* = 0.003), and 2014 (t = 4.98, *P* < 0.001).

### 7.7 Hematocrit levels

Lapland longspur hematocrit levels were significantly affected by sex, year, and their interaction (Table 3, Fig. 3). For both sexes, hematocrit levels were lowest in 2013 compared to all other years (Tukey’s HSD *P* < 0.05). Female hematocrit was lower than males in 2013 only (t = 5.10, *P* = 0.01).

White-crowned sparrow hematocrit levels were significantly affected by year but not sex (Table 3, Fig. 3). Hematocrit levels were significantly lower in 2013 compared to both 2011 and 2014 but not 2012 (Tukey’s HSD *P* < 0.05).

## 8 Discussion

Snow cover during the arrival period was higher in 2013 than all other study years at our field sites (Boelman et al., in review), and this pattern of prolonged snow cover extended across Alaska and parts of Canada as well (Lee et al., 2015). Both species migrate from the wintering grounds at lower latitudes through taiga and tundra habitats until reaching their breeding grounds. As a result of the extensive snow cover and cold temperatures, both Lapland longspurs and white-crowned sparrows arrived later on the breeding grounds by approximately 5 and 4 days, respectively (Boelman et al., in review). This late arrival could be attributed to harsh conditions en route that slowed migration by impairing stopover refueling as well as conditions in the vicinity of the breeding grounds that prevented birds from arriving (Sandberg and Moore, 1996, Newton, 2006, Boelman et al., in review). Anecdotal evidence from the popular press, suggests that in southeast Alaska, flocks of songbirds and waterfowl were seen in unusually large numbers, suggesting that birds were unable to move further north due to inhospitable conditions (Peluso, 2013).

Contrary to our hypothesis, baseline corticosterone concentrations were not different across years for either species. Short-term events in the wild, such as storms, have been found to cause acute elevations in baseline corticosterone (Wingfield et al., 1983, Rogers et al., 1993, Smith et al., 1994, Astheimer et al., 1995, Raouf et al., 2006, Jenni-Eiermann et al., 2008) and in captivity in response to food reduction or removal (Lynn et al., 2003, Lynn et al., 2010, Lendvai et al., 2014). Failure to detect difference in baseline corticosterone across years is surprising because it is likely that the birds in our study experienced metabolic challenges in 2013 as reductions in body condition were observed in both species. However, it is possible that in 2013 changes in HPA axis signaling were achieved through other mechanisms. For instance, in captive studies during short-term fasting, baseline concentrations of total corticosterone can increase rapidly while in response to long-term fasting reductions in corticosterone binding globulin (CBG) can result in the elevation of free corticosterone concentrations (Lynn et al., 2003, Eikenaar et al., 2014). Thus the elevations in baseline concentrations of free corticosterone during fasting, achieved through reductions in CBG, provide an alternative mechanism by which the captive birds continued to respond physiologically to food restriction. Alternatively, changes in sensitivity could have been enhanced through changes in receptor expression or 11β-hydroxysteroid dehydrogenase (11 β HSD) in brain and/or in peripheral tissues which may have allowed for static baseline concentrations of corticosterone (Breuner and Orchinik, 2001, Krause et al., 2015b, Lattin and Romero, 2015). Although we did not measure free corticosterone concentrations, our study birds may have responded similarly to the prolonged harsh conditions of 2013. This is the first study to describe baseline concentrations of corticosterone during a prolonged extreme event in our study region.

In partial agreement with our hypothesis, acute restraint stress in 2013 resulted in higher HPA axis activity compared to the other years of the study in Lapland longspurs, but was unaffected in white-crowned sparrows. Existing evidence suggests a positive relationship between stress-induced concentrations of corticosterone and environmental harshness in Puget Sound (*Z.l. pugetensis*) and Gambel’s white-crowned sparrows, bush warblers (*Cettia diphone*), Lapland longspurs, and snow buntings (*Plectrophenax nivalis*) (Wingfield et al., 1995, Addis et al., 2011, Krause et al., 2015a, Walker et al., 2015). The increase in HPA axis activity may be a product of reductions in food intake as shown in black-legged kittiwakes (Kitaysky et al., 1999) and in snow petrels (*Pagodroma nivea*) (Angelier et al., 2015). Current theory predicts that as the disturbance to homeostasis increases, HPA axis activity (e.g., corticosterone concentration) must increase so as to return to homeostasis (Angelier and Wingfield, 2013, Wingfield et al., 2015). We offer two possible explanations for why the HPA response of Lapland longspurs, but not white-crowned sparrows, was elevated in 2013. First, differences in conditions experienced en route traveling along the different flyways could have caused carry-over effects leading to elevated HPA axis activity in Lapland longspurs but not white-crowned sparrows. Second, Lapland longspurs prefer tussock dominated tundra, where exposure to the harsh conditions may have been greater than those experienced by white-crowned sparrows that can utilize microclimates within shrubs to avoid the worst of extreme conditions as demonstrated by greater wind attenuation (Wingfield et al., 2004). In addition, differences in availability or type of food between the two habitat types may have existed (Wingfield et al., 2004, Boelman et al., 2015). The combination of environmental factors may have been sufficient to cause an elevation of HPA axis activity in only one species. Responses of the HPA axis to stress can differ between species through differences in coping strategies or modification of physiological processes and as a result a clear pattern of endocrine or morphological response may not always be present (Dickens and Romero, 2013).

We also found that integrated corticosterone concentrations were significantly negatively correlated with mass, fat stores, pectoralis muscle profile and hematocrit levels in male (but not female) Lapland longspurs. Additionally female (but not male) white-crowned sparrows showed a relationship between integrated corticosterone and mass, only. These sex specific relationships suggest that one sex of a given species may be more susceptible to extreme events than the other. For example, male Lapland longspurs tend to arrive earlier on their breeding grounds relative to females, which results in a longer interval during which males are exposed to the harsh conditions. In female white-crowned sparrows, corticosterone may be regulating fuel metabolism and acting to suppress the reproductive axis through the activation of the Gonadotropin Inhibitory Hormone (GnIH) signaling pathway (Osugi et al., 2004, Ubuka et al., 2012).

Poor environmental conditions are known to cause declines in fat stores in birds (Wingfield, 1985, Sandberg and Moore, 1996, Martin and Wiebe, 2004, Newton, 2006). In agreement with our hypothesis, body condition in both of our study species was significantly reduced by the extreme conditions in 2013 compared to the other study years. Reductions in overall condition may be attributed to several factors such as reductions in food availability due to extensive snow cover since both species forage on the previous summer’s berries and seeds, as well as on arthropods, which only become available when patches of ground become snow free (Custer and Pitelka, 1978, Norment and Fuller, 1997). In addition, the low temperatures which prevailed during arrival likely resulted in compensatory elevation in basal metabolic rate to maintain core body temperature (Kendeigh, 1969). As a consequence of reduced food availability and higher metabolic rates, observed fat stores were low in Lapland longspurs during 2013 compared to 2011 and 2012, although not different from 2014. Interestingly, fat stores were higher in white-crowned sparrows in both 2013 and 2014 relative to other study years. A series of late-May snowstorms occurred in 2014 that may have had some effect on fat stores. Male white-crowned sparrows, under mild environmental conditions, are known to decrease fat stores as they establish territories following migration (Wingfield and Farner, 1978, Krause et al., 2015a), suggesting that in our study, white-crowned sparrows remained in migratory-like condition by maintaining higher fat stores, allowing them to better cope with harsh conditions (Sandberg and Moore, 1996, Ramenofsky and Wingfield, 2006). Although Lapland longspurs also decrease fat levels upon becoming territorial, they appear to lose fat at a slower rate than white-crowned sparrows (Krause unpublished data). This species specific response suggests a divergence in fat regulation between the two species in response to inclement weather. The long-term consequences or carryover effects of a harsh spring remain to be further investigated. Presumably initiation of breeding in a poor body condition could have negative effects of reproductive output as resources were devoted towards self-maintenance at a time in which birds are in preparation for breeding.

Similar to body fat stores, pectoralis muscle profiles in both species were significantly lower in 2013 compared to all other study years. It is important to note that muscle profile in white-crowned sparrows is largest during the arrival period and subsequently declines as birds become parental and then molt (Krause et al., 2015a). The reduced muscle profile may be attributed to protein deficiency in the diet, or overall dietary insufficiency which leads to muscle catabolism which may be primarily mediated through increased corticosterone signaling (Landys et al., 2006). Body mass was reduced in Lapland longspurs in 2013 relative to the other years of the study, but not in white-crowned sparrows. The reduction in body mass in Lapland longspurs may be attributed to the observed decline in muscle profiles and fat stores. A similar decline in body mass was observed in white-tailed ptarmigan (*Lagopus leucurus*) in an extreme year with extremely late snow cover (Martin and Wiebe, 2004). The failure to detect a difference in body mass in white-crowned sparrows may be caused by the large increase in their fat stores offsetting the reduction in muscle mass. Surprisingly there is almost a complete lack of data in the literature describing the changes in the body mass of any taxa in response to prolonged extreme events in the wild.

As hypothesized, hematocrit levels were reduced in Lapland longspurs and white-crowned sparrows in 2013 relative to other study years. Hematocrit levels are typically elevated during the arrival period since they are associated with periods of high metabolic activity (Fair et al., 2007, Krause et al., 2016a). In conjunction with the declines observed in other body condition, this result suggests that reduced hematocrit is associated with overall reductions in body condition. Nutrient and food deficiencies can lead to declines in hematocrit (Piersma et al., 2000), and such reductions have also been linked to reduced body mass (Acquarone et al., 2002). Interestingly, cold weather increases metabolic rate which necessitates an increase in oxygen carrying capacity of the blood and as a result hematocrit levels are elevated (Fair et al., 2007, Buehler et al., 2012). During migration to their arctic breeding grounds in 2013, our study birds were exposed to conditions that were colder than normal (Lee et al., 2015), but the birds arrived later in 2013 relative to other study years so that temperatures were not different at the time of capture among study years. The reductions in hematocrit in both species – in conjunction with reduced muscle profiles – suggest that reduced protein intake may have resulted in tissue catabolization and slowed red blood cell production.

## 9 Conclusion

The extreme weather conditions observed in 2013 caused major disruptions to spring events in both study species. To our knowledge, this is the first study to describe how the physiology and body condition of two species of arctic-breeding songbirds respond to extreme environmental conditions such as extended snow cover. Overall body condition was reduced in 2013 compared to the other study years, although we cannot untangle the ultimate causes of this decline as it could be attributed to conditions either on the breeding grounds or during migration. HPA axis activity was significantly increased in 2013 relative to other study years in Lapland longspurs only, which countered our hypotheses. This increase in HPA axis activity may be crucial for surviving the harsher condition experienced in 2013. Climate change models predict an increase in extreme events in the future which will test the limits of how species will cope. The exact coping mechanisms will likely be species specific but evidence from this study would suggest negative consequences on body condition while inducing compensatory changes in physiology.

## Author contributions

NB, LG, and JCW originally formulated the idea, field work and data collection were conducted by JSK, JHP, HEC, KEH, SKS, and SLM; data analyses were performed by JSK and JHP; the manuscript was written by JSK with critical input from all coauthors; and major funding was provided by NB, LG, and JCW.

## Figures and Tables

**Table 1:** Linear mixed effects models investigating the effects of year, restraint stress, and sex on circulating concentrations of corticosterone in Lapland longspurs and white-crowned sparrows. Bold text indicates significant result.

Independent variable	Lapland longspurs			White-crowned sparrows		
	df	*F*	*P*	df	*F*	*P*
Year	**2,58**	**5.87**	**0.005**	3,49	1.20	0.32
Sex	1,41	0.73	0.40	**1,46**	**4.08**	**0.05**
Mean air temperature	1,43	0.12	0.73	1,46	1.59	0.21
Snow cover%	1,44	0.29	0.59	1,46	2.74	0.10
Restraint	**3,30**	**119.83**	**<0.001**	**1,49**	**398.28**	**<0.001**
Year x restraint	**6,39**	**6.43**	**<0.001**	3,49	1.42	0.25

**Table 2:** The relationships among integrated corticosterone concentrations and fat stores, pectoralis muscle profiles, and hematocrit and in male and female Lapland longspurs and white-crowned sparrows. Bold text indicates significant result.

Species	Sex	Fat stores		Muscle profile		Mass		Hematocrit	
		R^2^	*P*	R^2^	*P*	R^2^	*P*	R^2^	*P*
Lapland Longspurs	M	**0.38**	**0.007**	**0.28**	**0.028**	**0.50**	**0.001**	**0.13**	**0.07**
	F	0.03	0.55	0.003	0.83	0.10	0.25	0.006	0.78

White-crowned sparrows	M	0.04	0.34	0.02	0.56	0.003	0.77	0.11	0.11
	F	0.25	0.25	0.10	0.48	**0.66**	**0.02**	0.04	0.69

**Table 3:** Linear mixed effects models investigating the effects of year and sex on total fat stores, pectoralis muscle profile, body mass, and hematocrit in Lapland longspurs and white-crowned sparrows. Bold text indicates significant result.

Variable	Lapland longspurs						White-crowned sparrows					
	Fat stores			Muscle profile			Fat stores			Muscle profile		
	df	*F*	*P*	df	*F*	*P*	df	*F*	*P*	df	*F*	*P*
Year	**3,263**	**4.53**	**0.004**	**3,263**	**25.63**	**<0.001**	**3,162**	**6.23**	**0.002**	**3,162**	**6.36**	**<0.001**
Sex	**1,263**	**6.49**	**0.01**	1,263	2.33	0.12	**1,162**	**3.86**	**0.05**	1,162	0.96	0.32
Year x Sex	3,263	0.43	0.73	3,263	0.53	0.65	2,162	1.16	0.31	2,162	1.25	0.28

Variable	Body mass			Hematocrit			Body mass			Hematocrit		
	df	*F*	*P*	df	*F*	*P*	df	*F*	*P*	df	*F*	*P*
Year	**3,263**	**6.04**	**<0.001**	**3,263**	**24.99**	**<0.001**	3,162	0.32	0.81	**3,162**	**6.62**	**<0.001**
Sex	**1,263**	**59.09**	**<0.001**	**1,263**	**4.39**	**0.04**	**1,162**	**39.95**	**<0.001**	1,162	1.30	0.26
Year x Sex	3,263	1.70	0.17	3,263	**4.05**	**0.008**	2,162	1.01	0.37	2,162	0.91	0.41
